# Supplementary material for: Are carbon emissions trading and green financial instruments synergistic? -Comprehensive quantitative research based on content analysis
Source: PLoS One. 2024 Mar 7;19(3):e0298601. doi: 10.1371/journal.pone.0298601 (PMC10919601; doi:10.1371/journal.pone.0298601)
Supplement: S1 Data — (ZIP) [file pone.0298601.s001.zip › Data/地级市Z_F工作报告.zip!/║┌┴·╜¡/╥┴┤║╩╨/└┤╫╘╣1⁄2╓┌║┼í╛┬φ┐╦╩2╛▌═°í┐_2017.docx]

各位代表：

现在，我代表市政府向大会报告工作，请予审议，并请市政协委员和其他列席会议的同志提出意见。

一、过去五年及2016年工作回顾

过去五年，是伊春发展史上极不平凡的五年。面对转型发展中的各种矛盾和困难，在市委的坚强领导下，在市人大、市政协的有力监督支持下，我们紧紧依靠全市人民，团结一心，开拓进取，攻坚克难，圆满完成了市十三届人大各次会议确定的目标任务，经济社会发展取得长足进步。一是立足全局保生态，森林资源质量数量同步提高。深入实施“天保二期”和《大小兴安岭林区生态保护与经济转型规划》，全面停止天然林商业性采伐，坚决落实严管资源各项措施，实现连续13年无重大森林火灾，森林覆盖率由83.9%提高到84.4%，林木蓄积量由2.64亿立方米增加到3.02亿立方米，首批跨入国家生态文明先行示范区行列。二是全力以赴促转型，新旧发展动能接续转换。积极应对全面停伐和过剩产能调整带来的巨大冲击，努力发展优势替代产业，成功引进中铁、华能、汇源等一批战略投资者，森林生态旅游、森林食品业增加值占GDP比重已达26%。“钢木经济”占GDP比重由2011年的16.3%降至4%，三次产业结构由32.6∶37∶30.4调整为41.3∶18∶40.7，多点支撑、多元驱动的产业格局正在形成。三是积极稳步抓改革，发展活力动力日益增强。以林业综合改革为统领，分类推进五营、新青、铁力林业局改革试点，44个贮木场、苗圃等林业附属单位转制成实体公司。圆满完成光明集团股改重组。对俄合作实现由木材采伐向经营林地、农业开发等多领域拓展，林区改革开放持续深化。四是高标定位建名城，森林城市功能品位全面提升。坚持高起点规划、高标准建设、高质量管理、高效益经营，扎实开展国家卫生城、文明城等重大载体创建活动，成功举办第十三届省运会，“三供三治”、公园广场等城乡基础设施建设进一步加强。总投资30亿元的华能伊春热电厂投入使用，居民供热和环境质量得到改善。森博会、国际森林马拉松等重大展会赛事品牌叫响，绿色伊春的知名度和影响力不断扩大。五是倾情倾力惠民生，城乡居民生活质量明显改善。民生投入力度加大，占公共财政支出的比例由61%提高到75%。实施了全省最大规模的林业棚改，总面积1846万平方米，惠及林区百姓75万人，6.6万富余职工实现转岗就业，林业职工工资提高1.5倍，“大集体”“五七工”等历史遗留问题陆续解决。相继获得国家卫生城、幸福城市及龙江首个“长安杯”等诸多殊荣，人民群众获得感持续增强！六是转变作风强服务，政府自身建设切实加强。扎实开展群众路线教育实践活动、“三严三实”专题教育、“两学一做”学习教育，从严整治“四风”。深入推进依法行政，大力简政放权，成立了市行政资源中心和企业投诉中心，完成市级统一规范权力清单工作，累计取消下放暂停行政审批544项，行风政风明显好转。

刚刚过去的2016年，是伊春发展史上具有里程碑意义的一年。在转型发展的关键节点，习近平总书记亲临伊春考察调研，为我们指明了振兴发展的方向和路径，更加坚定了走好绿色化转型发展新路的信心和决心。我们全面贯彻落实总书记重要讲话精神，坚决执行省市委决策部署，全力以赴推进保生态、稳增长、促转型、推改革、惠民生等重点工作。

——经济运行企稳向好。预计全年地区生产总值完成252亿元，同比增长1%，结束连续两年的负增长；固定资产投资完成76.7亿元，同比下降24%；公共财政预算收入完成14亿元，同比下降1.5%；社会消费品零售总额实现119亿元，同比增长10.5%。虽然受宏观经济下行、国家产能政策调整以及自身停伐压力仍在持续等多重因素影响，部分经济指标未能达到预期，但通过对重点领域、重点企业深化改革，挖潜增效，一批存量项目陆续转化为经济增量的效果逐步显现，一些主要指标降幅明显收窄，呈现稳中向好的积极变化。

——产业升级步伐加快。森林生态旅游业持续升温。创建全域旅游示范区全面启动，桃山庄四季温泉假日酒店、金水湾水上乐园等一批项目投入运营，中医药养生、湿地游、民俗民宿等新业态加速发展。“森林生态旅游节”和“森林冰雪欢乐季”系列活动全面铺开，冬季旅游产品不断丰富，首届伊春冰雪旅游产业国际峰会成功举办，“冰天雪地也是金山银山”这篇文章正在破题。全年入境游客首次突破千万人次，旅游收入突破80亿元，增幅均达40%以上。森林食品业链条不断拓展。制定出台了《关于加快推进林业产业发展的实施意见》，蓝莓等小浆果种植面积达6.6万亩，食用菌规模达5.1亿袋，森林猪饲养量达25万头，森林食品规上企业达38户。北药业稳步发展。平贝、返魂草等骨干品种规模持续扩大，喜人、东鹿等新建药厂已试产，种植、改培药材47万亩。木业加工创新提升。家具和木制工艺品产销两旺。光明家具营业收入增长两成，线上签单成交2.2亿元。美江、永达等企业主打的“北沉香”系列产品，年产20余万件。顺利通过“中国木艺之乡”复评。绿色矿业有序发展。鹿鸣钼矿稳定生产，实现产值9亿元。西钢集团经营回暖，实现产值25亿元。与上海海派玉雕文化协会务实合作，桃山玉、北红玛瑙品牌价值凸显。重点园区建设与“180招商计划”同步进行，21个项目落地开工，铁力、西林园区晋升为省级经济开发区，翠峦园区晋升为全省唯一的生态经济开发区。

——生态建设扎实推进。森林资源专项治理百日行动有效开展，查处各类林政案件149起，收回林地11公顷。翠北湿地、碧水中华秋沙鸭保护区晋升国家级自然保护区。完成更新造林28.6万亩，中幼龄林抚育293万亩。“伊春红松国家公园”体制试点方案完成编制上报。环境空气质量综合指数位居全省第一。

——改革攻坚取得突破。制定了《伊春重点国有林区改革方案》。政企合一区（局）公务员登记全面推进。机关事业单位养老保险制度改革顺利开展。公务员职级与职务并行改革启动推行。公车改革分步实施，取消公务用车361辆。伊林、旅游、供热三大集团相继组建，在体制机制创新上迈出实质性步伐。

——美丽伊春加速建设。改造棚户区1.15万套、农村危房1526户、老旧小区17个。完成既有建筑节能保温118万平方米。伊春火车站改造完成，伊翠铁路改线主体完工、开通使用。新建改建农村公路134公里。中心城净水厂全面建设，23座新建垃圾压缩中转站投入运营，医疗废物处理厂改造完成，山河大道等主要街路景观绿化一新。被列入国家新型城镇化综合试点地区。

——民生福祉持续提升。15件民生实事有效落实。全年城镇新增就业2.2万人，城镇登记失业率低于省控指标。12.3万厂办大集体人员纳入企业养老保险。义务教育学校标准化达标率、高考本科二表以上入段率分别高出全省25个和8个百分点。取消所有公立医院药品加成，林业中心医院被评为“全国百姓放心示范医院”。突出精准脱贫，分类实施“五个一批”工程，开展产业扶贫项目24个，落实资金3928万元，9809人实现脱贫，阶段性目标如期完成。

各位代表，五年来，我们在压力下加快了转型步伐，在实干中取得了新的业绩，发展成效令人鼓舞，砥砺前行让人铭记。这是省委、省政府和市委正确领导的结果，是市人大、市政协和社会各界鼎力支持的结果，是全市人民齐心协力、拼搏进取的结果。在此，我代表市政府，向全市人民及所有支持、参与伊春建设的各界人士，表示衷心的感谢和崇高的敬意！

成绩来之不易，经验弥足珍贵。回顾五年的发展历程，我们深深体会到，推动伊春的转型发展，必须紧紧依靠市委的坚强领导，保持政治定力，确保中央和省市委决策部署落地生根；必须以生态为先，打牢生态基础，厚植生态优势；必须以发展为要，大力发展优势特色产业，坚定不移走绿色化转型之路；必须以改革为重，全面消除体制机制束缚，向改革创新要活力；必须以民为本，尽心竭力改善基本民生，让林区群众生活得越来越好。

同时，我们也清醒地看到，伊春转型发展还存在诸多困难和问题：经济总量偏小，发展速度相对缓慢，生态资源的核心竞争力远没有释放出应有的效应；经济结构不尽合理，正处在新旧动能转换的关键阶段，产业优化升级的需求非常迫切；制约经济社会发展的体制机制障碍尚未从根本上消除，市场取向的改革任务艰巨；城市基础设施和基本公共服务体系还不够完善，一些涉及百姓切身利益的民生问题还没有彻底解决；政府职能转变尚不到位，部分公务人员服务意识不强、工作作风不实，营商环境有待进一步改善。对此，我们将高度重视并作为本届政府的工作方向，果敢应对，努力破解！

二、今后五年重点任务和2017年主要工作安排

按照市十二次党代会提出的主要奋斗目标，今后五年政府工作将紧紧抓住以下五项战略重点：一是科学保护经营森林，在打造生态文明先行示范区上实现新突破。牢固树立“生态就是资源，生态就是生产力”的发展理念，坚持保护优先，严守生态保护红线，确保森林总量不逆转、生态环境不破坏。科学经营森林资源，促进资源良性增长，活立木总蓄积达到3.64亿立方米，森林覆盖率稳定在85%左右，着力建设木材战略储备基地，重点保护好红松资源，打造中国最好的生态功能区，让伊春的天更蓝、山更绿、水更清，生态环境更美好。二是加快优化产业结构，在经济转型升级上实现新突破。围绕供给侧结构性改革，扎实做好改造升级“老字号”、深度开发“原字号”、培育壮大“新字号”三篇大文章。坚持“林业经济林中发展、林区工业林外发展”，做大一产、做优二产、做活三产。着力建设“一都一城三乡”，即中国森林食品之都、国际森林生态旅游名城、中国林都北药之乡、中国实木家具之乡、中国木艺之乡，加快提高五大重点产业质量和效益，推动电子商务、会展物流、健康养老等现代服务业繁荣发展，提高二三产比重，促进经济结构逐步优化，产业层次不断提升，生态资源优势转化为经济发展优势。三是全面深化改革开放，在创新驱动发展上实现新突破。坚持把深化改革、扩大开放作为推动发展的强大动力。着眼于增强生态功能、激发转型发展活力和保障职工群众利益，全面推进国有林区综合改革，如期实现“四分开”。深化重点领域和关键环节改革，加快非公经济发展。加强科技创新，挖掘优势资源潜力，推动产业转型升级。主动融入“龙江丝路带”建设，扩大以对俄合作为重点的对外开放。四是深入推进新型城镇化，在统筹城乡协调发展上实现新突破。围绕建设国际森林生态旅游名城目标，调整优化城镇布局，集中发展中心城区和节点城镇，着力建设一批森林生态符号鲜明的旅游小镇。持续完善城乡基础设施和公共服务功能，深入开展“美丽伊春”“智慧伊春”建设，全面提升城乡环境品质，加快形成城乡一体化发展格局。五是持续推动民生改善，在增进人民福祉上实现新突破。大力发展社会事业，加大科技文教、卫生体育、住房交通投入力度，扩大就业，增加居民收入，强化社会保障，做到保基本、兜底线、促公平，解决好群众最关心最直接最现实的利益问题。坚决打赢脱贫攻坚战，确保全面建成小康社会一个都不掉队，让全市人民享有更多获得感和幸福感。

按照市委部署，今年政府工作的总体要求是：全面贯彻党的十八大和十八届三中、四中、五中、六中全会，中央经济工作会议，省委十一届八次全会、省委经济工作会议和市第十二次党代会精神，深入贯彻习近平总书记系列重要讲话精神，特别是对我省两次重要讲话精神和对伊春的特指要求，按照“五位一体”总体布局和“四个全面”战略布局，牢固树立新发展理念，坚持稳中求进工作总基调，坚持以提高发展质量和效益为中心，坚持以推进供给侧结构性改革为主线，抢抓新一轮振兴东北老工业基地战略机遇，创新实施“五大规划”，加快发展“五大重点产业”，全力做好生态保护、经济转型、林区改革、城乡建设、民生改善等重点工作，促进全市经济平稳健康发展和社会和谐稳定。

主要预期目标是：地区生产总值增长6%，固定资产投资增长6%，规模以上工业增加值增长1%，公共预算收入增长5%，城乡居民收入增长与经济增长同步。

实现上述目标，重点做好以下八项工作：

（一）切实提高生态建设质量。强化保护优先意识，以更高的标准、更严的要求和更实的举措加快建设生态文明先行示范区。

全面加强生态保护。坚持从严治林，严格落实防火、防盗、防垦、防掠青“四防”措施，加强森林病虫害防治。深入开展林地“三乱”整治，强化项目用地管理，加大农林交错地带执法力度，坚决遏制非法侵占林地现象发生。全面加强自然保护区建设。继续开展生态移民，完成已撤并林场（所）验收工作，新完成9个林场（所）撤并。完善森防“四网四化”体系，重点建设森林防火道路372公里、靠前驻防营房7处，完成航空灭火机场建设前期工作，确保不发生重特大森林火灾。制定生态建设蓝皮书，严格落实领导干部生态环境损害责任追究办法，建立健全生态考核评价制度，形成生态保护长效机制。

大力培育森林资源。高质量更新造林，加强林间空地、荒山荒地植被恢复，提高补植补造面积比例，全年更新造林13.5万亩，红松果材兼用林营造比例达50%以上。推动5万亩特色经济林建设。提高森林抚育作业质量，积极推进铁力、美溪、友好、红星森林经营试点工作，全年抚育中幼龄林270万亩。支持苗圃市场化经营，推动苗木花卉产业发展，产苗量达到1.2亿株。

抓好生态环境治理。严格落实环保主体责任，不折不扣抓好中央和省环保督查组反馈意见整改落实。着力推进污染防治，切实抓好大气、水、土壤等重点领域污染治理。实施最严格水资源管理，严守“三条红线”，积极推行“河长制”，加大河流、水源地保护力度。强化种养生态环境治理，修复生态系统。淘汰老旧车、黄标车4000辆，淘汰建成区10蒸吨以下燃煤锅炉186台，全年空气质量优良天数占比达95%以上。

（二）加大经济结构调整力度。坚持“扬长避短、扬长克短、扬长补短”，发挥比较优势，精准施策发力，深入落实“互联网+”战略，促进一二三产融合发展，进一步推进经济结构优化升级。

做大一产。着力构建现代农业“三个体系”，坚持走市场化道路，深入推进农业供给侧结构性改革，发挥寒地黑土优势，把增加绿色有机农产品供给放在突出位置，切实提高农业发展质量和效益。坚持标准化种植，绿色有机面积增加到290万亩，占耕地总量的80%。尤其是近150万亩森工多种经营用地，逐步整合土地资源，按照优良品种、生产规程、投入品使用“三统一模式”，集中连片种植，打响绿色有机品牌。面向市场优化农业种植结构，调减玉米16.8万亩，扩大绿色水稻和有机大豆、杂粮等高效作物种植面积。加强“质量伊春”建设，抓好农业“三减”，定期开展农产品质量检测，推广全程可追溯体系建设，确保“绿色有机”可控、可见、可信。制定生态畜牧业发展规划，扩大森林猪、林下牛、森林鸡、雪貂等特色品种标准化养殖规模，年内饲养量分别达46万头、10万头、40万羽和17万只。落实承包土地“三权分置”办法，推进农村土地确权登记和经营权流转，完善“龙头企业+合作社+农户（林户）”产业模式，力争建设生产经营型合作社20个、省级现代农机合作社4个。加强农田水利建设，推进汤旺河、呼兰河及中小河流治理，切实保障农业生产安全。

做优二产。按照“五个要发展”，重点做好“三篇大文章”，推动工业发展提质增效。改造升级“老字号”，鼓励冶金建材、林产工业企业采用新技术、新工艺进行产品升级，重点推进西林钢铁产品结构优化，优质钢材产能达到150万吨，支持北方水泥研发新型材料，推动家具和木制品企业研发生产时尚环保产品，推动传统优势产业向优质、特色和高端化发展。深度开发“原字号”，依托优势资源，以“粮头食尾”“农头工尾”为抓手，加快桦树汁产品开发、蓝莓蓝靛果花青素萃取、红松籽油及软胶囊加工、森林猪肉制品加工等项目建设，增强农产品、森林食品、肉制品等原材料精深加工能力，提高产品附加值。培育壮大“新字号”，提高企业技术创新能力，围绕森林食品、生物医药、北药加工开发新产品。落实完成千户科技型企业三年行动计划，培育销售收入500万元以上科技型企业5户，引导宝宇等企业建设工程技术研究中心，支持中盟围绕红松籽等森林食品深度研发，建立院士、博士后工作站，促进科技成果尽快转化成长为新产业。

做活三产。加快推动生态旅游以及养生养老、休闲度假等生活性服务业创新升级，积极发展会议会展、现代物流、电子商务、金融服务、文化时尚等生产性服务业。着力培育山庄经济、观光农业等新业态，提升旅游业服务水平和质量。支持汇源会展中心完善功能，做强会展经济。依托汇源物流园，打造物流产业链。推进电商产业园建设，支持枫尚、越橘、四宝等电商企业和微商发展。加快体育休闲产业发展，办好国际森林马拉松等10项重大赛事。深入挖掘恐龙、民俗、红松等文化元素，建设富有林区特色的文化创作基地，着力将《映像·伊春》打造成地域文化符号。优化金融生态环境，鼓励银行、保险、证券等金融机构创新金融产品。

（三）加快推动五大重点产业发展。以市场化、多元化、特色化为导向，深度开发绿水青山、冰天雪地“两座金山银山”，努力把优势资源转化成产品和服务。

森林生态旅游业，重点抓好市场化经营、四季产品刻画和黄金线路打造。加快推进“全域旅游示范区”建设。深入推动国有景区体制、机制和经营模式创新，培育壮大以旅游集团为核心的市场主体。围绕“蝴蝶形”总体布局，差异化、特色化开发“春赏花、夏避暑、秋观山、冬嬉雪”四季主题旅游产品。着力补短板，加快打造“林都雪城”，高质量推出冬季特色旅游产品，举办好森林冰雪微马、林海雪原穿越等品牌赛事，推动雾凇、温泉、亲子冬令营及民俗等产品关联开发。加强与国内大型旅行社、省内重点景区旅行社合作，串点成线，内引外联，融入全省旅游大格局。春季重点推出以红星火山岩地质公园、桃山悬羊峰为中心的“赏杜鹃花海”精品线路。夏季重点推出上甘岭溪水国家森林公园—五营国家森林公园—汤旺河林海奇石风景区—嘉荫茅兰沟风景区“呼吸中国好空气休闲度假”精品线路。秋季重点推出朗乡万松岩景区—碧水中华秋沙鸭自然保护区—凉水自然保护区“赏五花山摄影采风”精品线路。冬季重点推出桃山森林冰雪玉温泉—桃山国际狩猎场—铁力日月峡滑雪场—库尔滨雾凇“森林冰雪体验”精品线路。

森林食品业，重点抓好基地建设、产业链延伸和品牌塑造。统筹产业布局，扩大标准化基地建设规模，新建上甘岭等3个小浆果基地、新青等3个森林猪基地、翠峦等6个黑木耳规模化棚室基地。加快“红蓝黑黄”等产业链延伸，丰富下游产品，向精深加工要效益。深入实施商标战略，在发挥现有品牌效应基础上，加大整合力度，集中打造“伊林”“伊森林”“伊森工”品牌，叫响“伊春小兴安岭大森林”绿色有机森林食品。

北药业，重点抓好试验基地建设、标准化种植和就地加工。加强与大专院校、科研院所、中药企业合作，建设药材试验基地，开展品种驯化和示范栽培。按照国家GAP标准，坚持“单品种、规模化、标准化”，推动南岔、汤旺河、红星和铁力局等“森林百草园”建设，扩大平贝、刺五加、返魂草等骨干品种规模，北药种植改培达到50万亩。支持葵花、格润、金北药等龙头企业加快技改和产品研发，提高道地药材就地加工能力。推进“医养药游”一体化发展，加快南岔永翠河国际健康疗养院等建设，提升北药产业价值链。

木业加工业，重点抓好产品档次提升、龙头企业培育和市场开拓。坚持中高端定位，推进光明工业园二期和双丰家具园建设，支持光明、双丰、友春等龙头企业带动中小企业联合发展，开发环保实木家具。推动以“北沉香”为重点的根雕木艺产业发展，扶持壮大美江、永达、侨艺等龙头企业，开发特色旅游纪念品。加大实体连锁店和网上旗舰店建设力度，拓展线上线下营销渠道，针对特定人群开展家具订制、高档家装业务，开拓大城市市场，叫响“实木家具之乡”和“木艺之乡”，重塑传统产业竞争发展新优势。

绿色矿业，重点抓好现有企业达产达效、产业链延伸和非金属矿产资源高效利用。坚持开发一点、保护一面、带动全局，有序推动矿业经济发展。推动鹿鸣钼矿围绕产能释放，强化企业管理，积极推动钼金属和尾矿综合利用，实现产值13亿元。推动西林钢铁加快战略重组，支持翠宏山铁矿和五星石灰石矿等原料基地建设，加强钢渣开发利用。努力增产，实现产值50亿元。帮助北方水泥拓展市场、扩大销售，力争销售水泥100万吨。跟踪省地质勘查三年专项项目，掌握贵重金属矿种勘查结果，争取国家林地占补平衡政策，获得矿权择机开发。推进地热温泉等矿产资源开发与利用。集约开发玛瑙玉石资源，着力提高“北红玛瑙”“桃山玉”产品档次、品牌价值以及对文旅产业的助推效应。

（四）深入实施产业项目建设。坚持把项目建设作为支撑产业发展、结构调整、经济转型的重中之重，全年安排省市级重点产业项目44个，总投资191亿元，年度计划投资35亿元。

推进投产项目达产。从严落实落靠项目包保责任，一企一策地解决投产企业水电热、资金、运输等生产瓶颈问题。重点推进中盟红松球果全产业链建设、黑尊黑木耳菌包生产、永旺食品大豆精深加工及鹿鸣钼矿等6个已投产重点项目全面达产达效。加快推进喜人、天一和东鹿制药等已试产企业尽快全线生产。支持华能伊春热电厂新上固体电蓄热调峰项目，帮助争取增加上网电量，力争达到全省平均发电小时数。推动廻龙湾职工休养中心尽早运营。

推进在建项目投产。加大建设力度，确保按计划完成投资进度和形象进度。加快推进汇源绿色产业谷、忠芝即食木耳、高锶矿泉水等森林食品项目，嘉荫滨江旅游带精品景区、歧黄养老养生苑、带岭敦豪度假村、桃山狩猎部落等生态旅游项目，以及铁力兴叶家具、乌伊岭北红玛瑙玉石文化产业基地、升辉农贸产品综合批发市场等项目建设，全年在建重点项目力争投产19个以上。

推进招商项目落地开工。坚持绿色发展理念，坚决不上破坏生态环境的项目。大力开展实效招商，争取引进辐射、带动作用强的大项目6个以上。紧跟线索，推动签约，重点加大中航航空机场、大庄园森林食品研发、莲花集团旅游综合开发等重大项目对接跟进力度。推动金新农生猪生态养殖、九河泉生态农业产业化、新青旅居养老小镇、金林矿业铅冶炼等16个项目开工建设。

抓好项目谋划储备。围绕国家政策导向、五大重点产业和优势资源，拓展项目源，丰富项目库。全年谋划能够带动产业链上下游互动发展的重大项目125个以上，其中，市级不少于20个，每个县（市）区（局）不少于5个。

提升园区承载能力。重点完善省级经开区基础配套设施。推进翠峦园区建设“三年攻坚战”，开工建设污水处理厂、3纵1横1桥道路网格及供热、供水、蒸汽管线等设施，可利用面积达70公顷，力争入驻企业30户。推进铁力园区提档升级，加快雨污排水管网建设，改造路面10万平方米，可利用面积达279公顷，力争年内产业项目完成投资10亿元。

（五）深化林区综合改革。积极稳妥推进重点领域改革，在体制机制上动真格，在更深层次上求突破。

重点推进国有林区改革。按照“四分开”要求，加快完善改革方案，明确改革方向和路径。推进企业办社会职能剥离，上半年完成“南四局”教育、医疗职能剥离，13个政企合一区（局）适时跟进。按照产权清晰、权责明确、政企分开、管理科学的现代企业制度要求，完善伊林集团、旅游集团、供热集团自主经营、自负盈亏的法人治理结构和运营模式，统筹注入优良资产，积极吸纳工商资本，推动企业尽快做大做强。推进林业局经营体制改革，加快林场所实体经济发展。推进公务员登记、林业管理机构分类和定岗定编工作，着重解决好人员分配、职能转移衔接配套问题。推动事业单位分类改革和森林公安体制改革。

持续推进关键环节改革。落实“三去一降一补”改革部署，全面完成去产能任务，着力降低实体经济企业成本。制定出台支持民营经济发展实施意见，支持各类社会资本通过PPP模式参与公共事业投资，大力发展非公经济。落实“营改增”及小微企业结构性减税政策，扩大“过桥贷款”“助保贷”业务范围。深化商事制度改革，推进“五证合一”和个体工商户“两证合一”登记制度落实。放宽经营场所登记条件，催生更多市场主体。

着力拓展对外开发开放。积极融入“龙江丝路带”建设，协调推动嘉荫口岸早日恢复通关，推进跨境产业园、对俄互市贸易区规划设计申报工作，鼓励林业局及社会企业赴俄开展林农开发。加强与日、韩、芬兰以及东北东部区域（12+2）等国际国内友好城市的联系交往，在产业发展、生态保护、科技文化等领域开展务实合作。巩固欧美、东南亚等传统市场，努力扩大合作领域和贸易规模。

（六）加强城乡建设管理。启动新型城镇化综合试点工作，统筹推进中心城区、区局城镇、场所乡村基础设施建设，完善市政与公共服务功能，加快建设“美丽伊春”。

提高城市规划水平。立足城镇集群和人口集聚，完成城市总体规划修编和控详规划近期建设范围全覆盖，加快“四区融合”。完成新区南区城市设计编制，抓好新建棚改区、旅游名镇和特色小镇规划设计。加快推动多规合一。突出规划的刚性约束，加强对道路节点景观、建筑造型、城市色彩的设计管理，保证城市风格协调，特色鲜明。

抓好基础设施建设。加快汤伊公路改扩建，确保明年年底通车。推进汤嘉公路建设前期工作，争取年内开工。投入2.3亿元，完成总长34公里的西岭至鹿鸣钼矿、五营至五营森林公园旅游防火公路建设，开工建设总长46公里的溪水至林都机场、五营至梅花山滑雪场旅游防火公路，推动带岭至丰林林场、平顶山至新兴玉石矿、溪水至青山道桥3条道路项目前期和资金争取工作。谋划伊春至铁力快速铁路项目，启动前期工作。支持乌伊岭至黑河沿边铁路建设。继续实施棚户区改造，提高货币化安置比例，消化存量房源。抓好廻龙湾大街、奋斗街等市政道路建设。加大供热改造力度，统筹推进中心城区供热资源整合、管线并网，支持华能主管网建设，新建和改扩建供热管网5000米。开工建设西林、朗乡垃圾处理厂，完成乌伊岭、红星、五营、浩良河镇污水处理厂建设并投入使用。加快推进管道燃气进户工程。抓好伊春河伊春段、翠峦段两岸及哈伊高速出口、锦绣山水公园绿化景观建设。完成伊翠铁路原线拆除工程，规划建设绿色休闲长廊。

提升城市管理水平。注重与旅游城市定位相适应，加强精细化、网格化管理。推进“智慧伊春”时空信息云平台建设。加强棚改新建区、弃管小区物业管理。加大环卫投入力度，支持中心城区新上保洁车12台。狠抓市容环境综合治理，突出抓好“三大市场”、商业步行街、客运站周边经营秩序整治，全力做好“创文”工作。

（七）更大力度保障和改善民生。坚持“转型发展，民生为要”，把民生工作放在财力投入首位，从事关群众切身利益的地方做起，让民生工作更有厚度、更有温度。

加快发展社会事业。推进公办幼儿园建设，新建6所标准化幼儿园。完成铁力市等6个县区义务教育均衡发展任务，确保在全省率先整体实现均衡发展目标。进一步调整优化高中学校布局，由现有的16所撤并到12所。强化省级重点高中教学管理，采取有效措施强化教师队伍建设，努力实现高考连增。加强职业院校建设，促进与产业园区、重点企业的产教融合、校企合作。稳步推进城市和县级公立医院改革。积极推进分级诊疗，提高基层医疗卫生机构服务能力。开展系列文化惠民活动，加快构建覆盖城乡的公共文化服务体系。推进“健康伊春”建设，开展全民健身活动。

加强社会保障。做好全民参保登记工作。推进厂办大集体人员纳入养老统筹后续工作。加快城乡居民基本医疗保险整合。继续提高低保标准，城市低保由每月500元提高到530元，农村低保由每年3500元提高到3710元。加强困难人群救助工作，健全完善社会救助救济体系，全面推行“救急难”制度。

推动全民创新创业。重点推动林业富余职工、大学生、科技人员“双创”。加强林业富余职工转岗培训，提升就业创业技能。推进翠峦小微企业园、电商产业园等孵化基地建设，放大大学生创业“种子资金”扶持效应。全市力争新增就业2万人。打通科技成果转化通道，支持市林科院、农研中心等科研院所与企业对接合作，鼓励专业技术人才兼职兼薪和离岗创业。打开“绿色通道”，积极引进转型重点产业紧缺人才和高技能人才。

维护社会和谐稳定。强化社会治理体系和治理能力建设，落实安全生产责任，抓好非煤矿山、危化、道路交通、建筑、消防、食品药品等重点行业领域安全监管，坚决杜绝重特大事故发生。落实信访工作责任，畅通群众利益诉求表达渠道，主动在基层和源头排查化解矛盾纠纷。抓好社会治安综合治理，依法打击各类违法犯罪活动，提高突发公共事件应急处置能力，提升“平安伊春”建设水平。

打好脱贫攻坚战。严格按照“六个精准”要求，推进扶贫工作有序开展。突出产业扶贫，用好用活4000万元专项扶贫资金，将第一批市级扶贫专项产业项目全部落实到位。抓好精准识别，明确贫困人口脱贫退出时序，年内实现18个省市级贫困村摘帽，3261户、5323人脱贫。

（八）加强政府自身建设。今年是新一届政府工作开局之年，各级政府要以更严的要求、更实的作风、更高的效能履职尽责，担当起加快转型发展的重任，建设人民满意政府。

坚持依法行政，建设法治政府。牢固树立法治意识、法治理念，自觉运用法治思维和法治方式，依法依规推动工作，做到法无授权不可为、法定职责必须为。坚持“放管服”结合，进一步简政放权，坚决落实国家和省精简行政审批事项，推动权力、责任“两清单”全覆盖。大力优化发展环境，深入开展优化投资营商环境专项行动，不断推进“法治伊春”“诚信伊春”建设。规范重大行政决策程序，加快政府法律顾问制度落实，全面推行政务决策、执行、管理、服务、结果“五公开”。强化权力监督制约，完善行政执法监督体系，自觉接受人大依法监督、政协民主监督和社会公众监督，让权力在阳光下运行。

改进工作作风，建设高效政府。要狠抓工作落实，坚持讲实话、办实事、求实效，对认准的事和决定的工作，要坚决干、一刻不停歇地干，切实把改革发展稳定各项任务压实落靠，说到就要做到，决不能放空炮。要担当尽责，大力倡导务实勤勉、敢于负责的过硬作风，哪里有困难就出现在哪里，哪里有问题就解决在哪里，哪里有矛盾就化解在哪里，切实提高执行力，坚决纠正不作为、慢作为等庸政懒政怠政行为。要加强学习，坚决按照党中央对经济形势和经济工作作出的重大判断、重大决策、重大调整，加快知识更新，高度关注和适应新常态下经济转型、改革深化的新要求，强化专业素养，提高工作专业化水平。要坚持问题导向和底线思维，以时不我待的责任意识，撸起袖子加油干，用铁的肩膀和过硬本领扛起干事创业的担子。

践行服务宗旨，建设为民政府。要坚持以人民为中心的发展理念，始终把增进人民福祉作为政府一切工作的出发点和落脚点。要加强调查研究，将工作重心下移、力量下沉，深入基层、深入群众，刹下心来解决群众反映强烈的困难和问题，真心实意为民解忧。要善于解剖麻雀，把实际情况摸准摸透，用正反两方面的典型推动工作，做到胸中有数、有的放矢。要坚持问计于民，用好“向人民报告，请人民监督”等民主评议载体平台，广泛推动网络问政，努力使各项工作最大程度地吸纳公众智慧、体现人民意愿。要优化服务，切实提高行政资源中心审批集中度，拓展网上政务中心服务内容，让服务流程更合理、更高效，让群众少跑腿、好办事、不添堵。

强化纪律约束，建设廉洁政府。严守政治纪律和政治规矩，切实增强“四个意识”，特别是核心意识、看齐意识，坚决维护以习近平同志为核心的党中央权威，坚决贯彻落实省市委决策部署。严格落实党风廉政建设“两个责任”，自觉执行《准则》《条例》和各项廉政规定，强化对公共资金、国有资源、国有资产等重点领域、关键环节的廉政风险防控和审计监督，对违法违纪和腐败行为“零容忍”。厉行勤俭节约，大力压缩行政运行成本，把有限的资金用在发展最急需的地方，用在民生最急迫的事上，做到“三公”经费“零增长”，以风清气正的政府形象凝心聚力、推动发展。

各位代表，发展要靠实干，努力奋斗才能梦想成真。推动伊春转型发展、全面建设小康社会的重任，历史地落在了我们这届政府身上，任务光荣而艰巨。我们既然担负神圣职责，就要不辱使命；我们既然身负组织重托，就要不忘初心；我们既然肩负人民信任，就要不懈努力。让我们紧密团结在以习近平同志为核心的党中央周围，在市委的坚强领导下，坚定信心，开拓创新，奋发有为，让伊春老林区焕发青春活力，书写好绿色化转型发展的新篇章，以优异成绩迎接党的十九大和省第十二次党代会胜利召开！
